# Appropriate time to apply control input to complex dynamical systems

**DOI:** 10.1038/s41598-020-78909-8

**Published:** 2020-12-16

**Authors:** Ali Ebrahimi, Abbas Nowzari-Dalini, Mahdi Jalili, Ali Masoudi-Nejad

**Affiliations:** 1 Laboratory of Systems Biology and Bioinformatics (LBB), Institute of Biochemistry and Biophysics, University of Tehran, Tehran, Iran; 2 School of Mathematics and Computer Science, University of Tehran, Tehran, Iran; 3 School of Engineering, RMIT University, Melbourne, Australia

**Keywords:** Network topology, Statistical methods

## Abstract

Controlling a network structure has many potential applications many fields. In order to have an effective network control, not only finding good driver nodes is important, but also finding the optimal time to apply the external control signals to network nodes has a critical role. If applied in an appropriate time, one might be to control a network with a smaller control signals, and thus less energy. In this manuscript, we show that there is a relationship between the strength of the internal fluxes and the effectiveness of the external control signal. To be more effective, external control signals should be applied when the strength of the internal states is the smallest. We validate this claim on synthetic networks as well as a number of real networks. Our results may have important implications in systems medicine, in order to find the most appropriate time to inject drugs as a signal to control diseases.

## Introduction

Applications of control theory is no longer confined to a small technical system and has been recently expanded into complex network systems^1–5^. The aim of a control system is to look for a sequence of interventions in the network, so that the state of the nodes is hierarchically changed to a desired reference condition^6,7^. Finding driver nodes is an essential step toward controlling a dynamical system. The study of network controllability and determining the minimum number of driver nodes^8^, has attracted much attention within the community of network science and dynamical systems^9–15^. Various applications have for network control technology^16–20^.

There is a rich literature on theoretical and empirical studies which have examined how a dynamical system responds to external signals^21–27^. The degree of flexibility of a dynamical system has been introduced and the interaction between the flexibility values of a dynamical system and the effect of an external signal applied to the network has been examined^22^. In another work, the effect of local divergence on the synchronization capabilities of paired oscillators as well as the effect of slow transition have been investigated and some of the dynamical systems’ conditions have been depicted, under which the highest ability to synchronize these oscillators could be achieved^24^. Moreover, concerning the connection between noise in a dynamical system and its stability, random intermittent stabilization based on discrete-time or time-delay feedback has been examined^25^. It has also been proved that a dynamical system stimulated by a noisy input with a time delay can produce the same effects as a dynamical system with a typical delay stimulated by the same noise^27^.

In a network system, the set of driver nodes with the minimal size is often not unique^28^. Choosing the best driver nodes to control have an active research topic within the community of network science^29,30^. To select a set of driver nodes to facilitate an optimal control of the network, one should further consider some important secondary issues, such as minimizing the amount of energy required for the control^5,31,32^, decreasing mediator nodes as well as the possible side effect of the control process^15^ and the number of control steps^33^.

Determining a set of driver nodes for an optimal control of a network is a necessary but inadequate action. Moreover, identifying the type of interventions, which should be applied to the network, is of the utmost importance. To minimize the energy required to control a network, it is crucial to determine the time that the inputs should be fed into the network such that the control performance is maximized.

As an example, in Bioinformatics, suppose we would like to input the signals into some effective agents to induce the apoptosis of cancer cells and eliminate this type of cells under minimum effort (energy)^34–36^. Taking the state of network variables and the internal fluxes into account, lack of knowledge about the best time for applying the external signals is a major challenge. As another example^37–39^, imagine a person would like to take control of a society or make some changes in the way a community is being led. If we assume that people are network nodes in this community, considering the fact that network nodes are under the influence of signals that agree or disagree to alterations in a particular subject within a population, thereby within their various communications, it is critical to find the best time when one can input the signals into the network. In this manuscript, we propose a methodology to determine the optimal time to apply the external inputs to driver nodes. We apply the proposed method on some real networks and reveal its effectiveness.

## Results

States of individual nodes in complex dynamical network systems often undergo continuous changes based on their interactions with other nodes. Our aim is to find the best time that an external input can be applied to nodes in order to have maximum control impact. Our methodology is based on the connection between the strength of the system’s internal fluxes and the effect of the external signals on the system. As an external signal is applied to the network, the existing interaction between network nodes and the adjacent systems is ignored and only the network’s internal connections are taken into account in order to calculate the study the relation between the strength of internal fluxes and effect of the external control.

Let’s consider a directed network $G$ with $n$ nodes with equations of motion described by the

1 $$\dot{\user2{X}}\left( t \right) = A{\varvec{X}}\left( t \right) + B{\varvec{U}}\left( t \right)$$

where $A$ is the connectivity matrix between the nodes, ${\varvec{X}}$ is the state vector, ${\varvec{U}}$ is the input vector and $B$ determines the driver nodes to which the external input should be applied. The solution to the above equation will be

2 $${\varvec{X}}\left( t \right) = e^{At} {\varvec{X}}\left( 0 \right)$$

if there are not any external signals, and

3 $${\varvec{X}}\left( t \right) = e^{At} {\varvec{X}}\left( 0 \right) + \mathop \smallint \limits_{0}^{t} e^{{A\left( {t - T} \right)}} B{\varvec{U}}\left( T \right)dT$$

if the external signals are applied to the network^40^.

In the Methods section, we prove a theorem which specifies that the maximum effect of input signals to control a system will be produced when the system’s internal fluxes are weak. A dynamical system will have strong internal fluxes when it is not in a steady-state mode or when the equilibrium of the system could be related to the positive and negative signals that are balanced. In such case, external control signals often have minimal effect on the system’s performance. Our proposed method is based on this principle.

Let’s denote the network internal signals of nodes by *h*(*t*). This function *is* differentiable at every point, when the left- and right-hand derivatives are equal, and the derivative is the degree of stimulation or the tendency for the movement of state of node. We know that the derivative of a function is zero if and only if the function is positioned at the extremum points, where the systems is its steady-state. The strength of the input signals is a function of the extent of movement in the preequilibrium, the duration of the stay in the equilibrium and the network structure.

To evaluate the above idea, here we present evidence on both synthetic and real networks. We compute the relationship between the strength of network’s internal fluxes and the degree of the effectiveness of external input signals on the network. Negative correlation coefficient between the strength of the internal fluxes and the effect of the external inputs denotes an inverse association between them. Our results show that when the strength of internal fluxes is high, the impact of external input signals on the network will be low, and vice versa.

As shown below, the functions $f\left( t \right)$ and $g\left( t \right)$, respectively, are employed to measure the strength of internal network fluxes and the degree of the effectiveness of external signals. The function $f\left( t \right)$ determines the sum of the derivatives of the states at time *t* by applying the following equation:

4 $$f\left( t \right) = \mathop \sum \limits_{n} \left| {\dot{\user2{X}}\left( t \right)} \right|\;{\text{s}}.{\text{t}}\;\left| {\dot{\user2{X}}\left( t \right)} \right| = \sqrt {Re^{2} \left( {\dot{\user2{X}}\left( t \right)} \right) + Im^{2} \left( {\dot{\user2{X}}\left( t \right)} \right)}$$

where *n* is the number of nodes and *Re* and *Im* are the real and imaginary parts of the complex number, respectively. The function $g\left( t \right)$ is obtained from the following equation:

5 $$g\left( t \right) = \mathop \sum \limits_{n} \left| {{\varvec{Y}}\left( {t + m} \right) - {\varvec{Z}}\left( {t + m} \right)} \right|$$

where ${\varvec{Z}}\left( {t + m} \right)$ specifies the state of the nodes without applying the external signals to the system after $m$ steps, and ${\varvec{Y}}\left( {t + m} \right)$ determines the state of the nodes by applying the signals to the system at time *t* after *m* steps.

Based on the states obtained in the previous step and the available signals, the state of network nodes is obtained as:

6 $${\varvec{X}}\left( t \right) = e^{A} {\varvec{X}}\left( {t - 1} \right) + \mathop \smallint \limits_{t - 1}^{t} e^{{A\left( {t - T} \right)}} B{\varvec{U}}\left( T \right)dT = e^{A} \left( {{\varvec{X}}\left( {t - 1} \right) + \left( {\mathop \smallint \limits_{t - 1}^{t} e^{ - At} dT} \right) \times {\varvec{U}}} \right) = e^{A} \left( {{\varvec{X}}\left( {t - 1} \right) + A^{ - 1} \left( {I - e^{ - A} } \right) \times {\varvec{U}}} \right)$$

and $e^{A} = M\hat{e}M^{ - 1}$ in which

7 $$M = \left[ {m_{1} \left| {m_{2} } \right| \ldots {|}m_{n} } \right] \;{\text{and}}\;\hat{e} = \left[ {\begin{array}{*{20}c} {\begin{array}{*{20}c} {e^{{\lambda_{1} }} } & 0 \\ 0 & {e^{{\lambda_{2} }} } \\ \end{array} } & {\begin{array}{*{20}c} 0 & 0 \\ 0 & 0 \\ \end{array} } \\ {\begin{array}{*{20}c} 0 & 0 \\ 0 & 0 \\ \end{array} } & {\begin{array}{*{20}c} \ldots & 0 \\ 0 & {e^{{\lambda_{n} }} } \\ \end{array} } \\ \end{array} } \right]_{n \times n}$$

where $\lambda_{1} ,\lambda_{2} , \ldots ,\lambda_{n}$ are eigenvalues and $m_{1} ,m_{2} , \ldots ,m_{n}$ are eigenvectors of *A*. In order for the matrices *M* and *A* to be invertible, ${\text{det}}\left( A \right) \ne 0,$ and *A* must have $n$ distinct eigenvalues.

**Figure 1:**
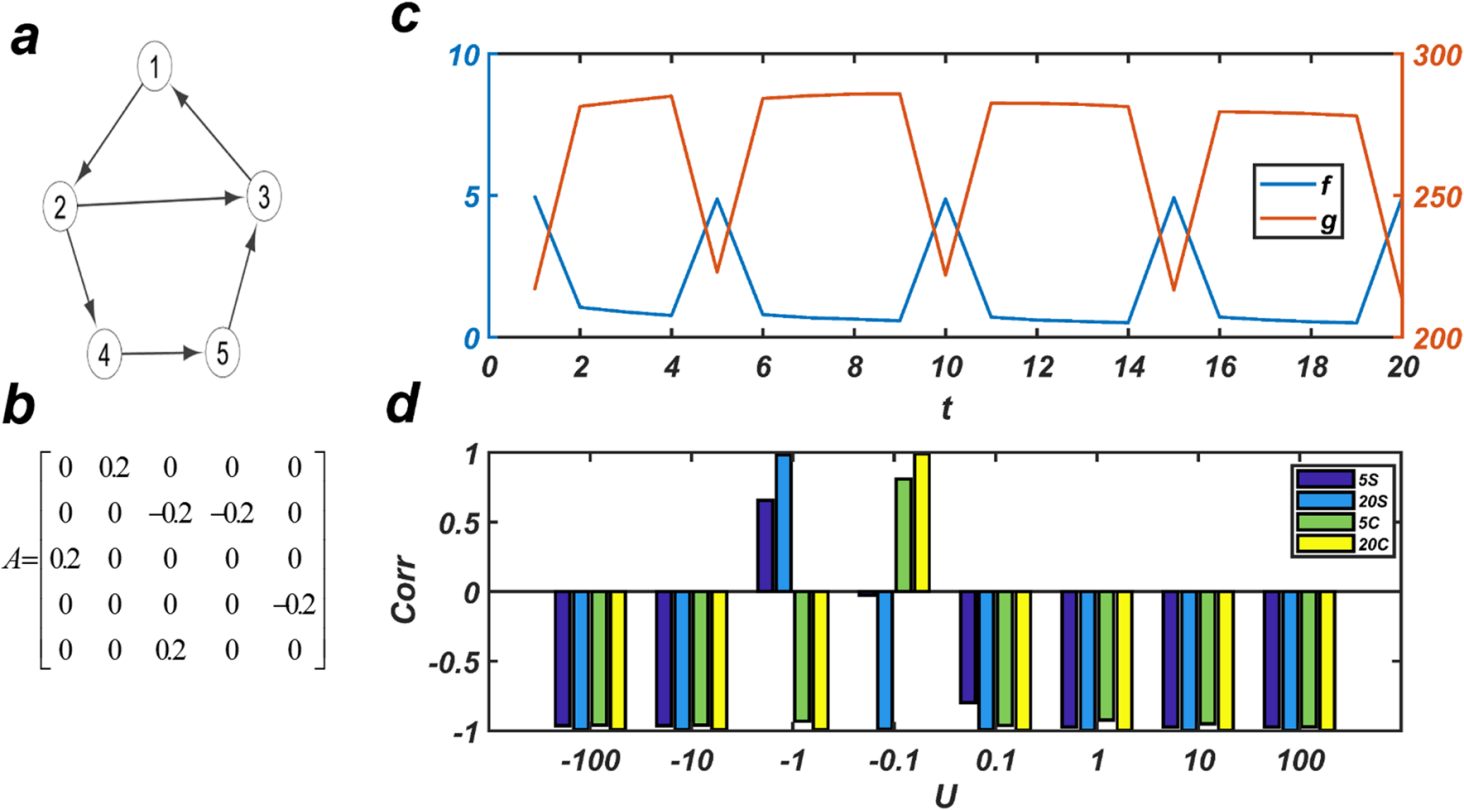
The results on a toy network: By applying different signals to the network, it is shown that there *f* and *g* are negatively correlated. (**a**) A sample directed network with 5 nodes and 6 edges. (**b**) Adjacency matrix of the network; the weights of edges are 0.2 and − 0.2. $\det \left( A \right) \ne 0$ and *A* has 5 distinct eigenvalues. **c)** The graph shows *f* and *g* in the interval $t = 1$ to $t = 20$. An input signal with a value of -1 is applied to the nodes in 20 consecutive steps (*m* = 20). The correlation coefficient between *f* and *g* is $- \;0.9917$. (**d**) The graph shows the correlation coefficient between *f* and *g* with different input signals as ${\varvec{U}} = - 100, - 10, - 1, - 0.1, 0.1, 1, 10, 100{ }.$ If a single signal is applied, then *m* = 5 *(*5*S)* and *m* = 20 (20*S*), respectively and if a consecutive signal is applied, then *m* = 5 *(*5*C)* and *m* = 20 (20*C*), respectively**.**

Figure 1 represents a topy network. We take the initial states of the nodes as $\left[ {1,1,1,1,1} \right]^{T}$ and the time intervals as $t = 1\;{\text{to}}\;20$. At times $t = 1,5,10,15,\;{\text{and}}\;{ }20,$ a signal with the value of 1 is applied to the nodes of the network. Thus, the amount of internal fluxes of the network at $t = 1,5,10,15{ }\;{\text{and}},20$ will be high; otherwise it will be low (blue curves). At any specific time, different input signals are applied to the network, and the correlation coefficient between *f* and *g* is calculated. There exist two strategies for applying the signal at time *t*. In the first strategy, the signal is applied at the first step, and there are not any signals in the next *m-*1 steps. While in the other strategy, a continuous signal with a length of *m* is applied to the network. The value of *m* is 5 or 20.

### Results on synthetic networks

**Figure 2:**
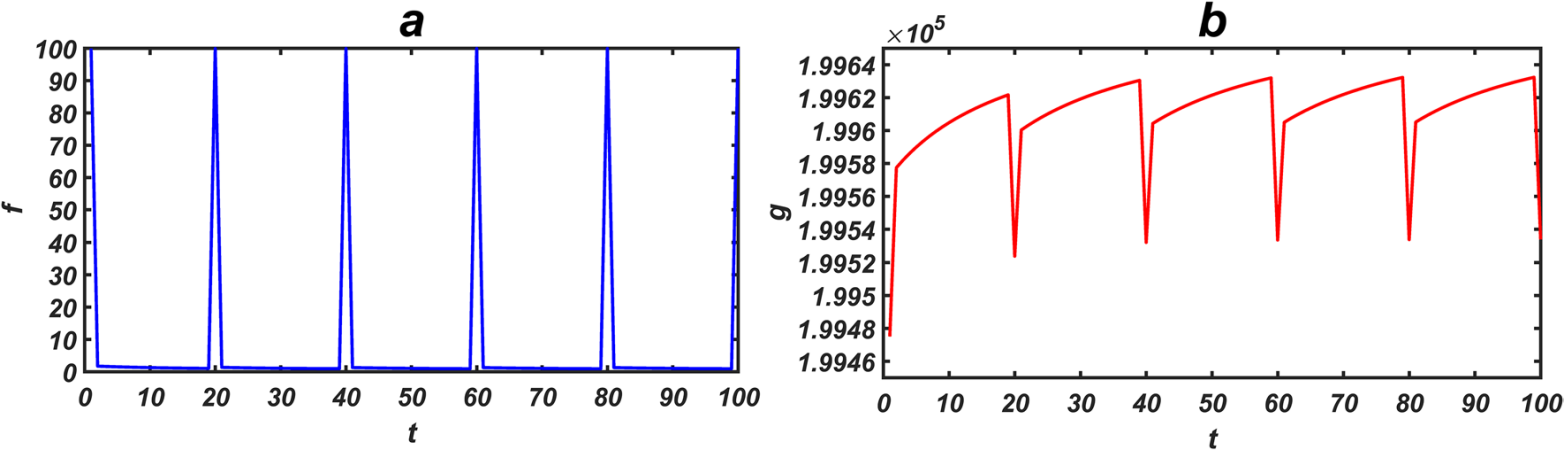
The graph shows *f* and *g* in a network with 100 nodes and average degree 〈*k* = 10〉. The initial value of the state of nodes is considered to be 1. (**a**) The horizontal axis represents the intervals from $t = 1$ to $t = 100$, where the input signal is applied to the network at $t = 1,20,40,60,80,100$. (**b**) The function *g* where an input signal with a value of -100 is to all nodes in 20 consecutive steps.

**Figure 3:**
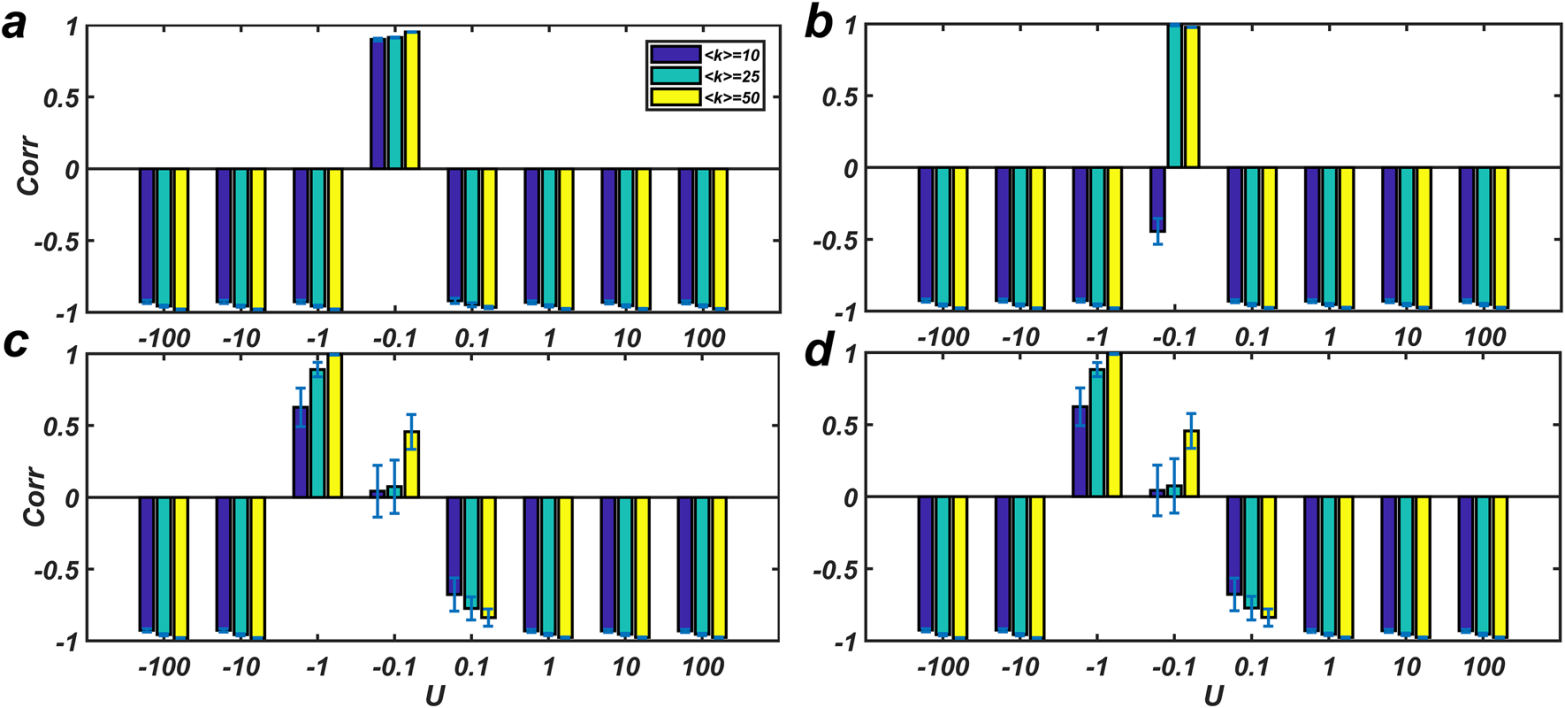
The correlation coefficient between *f* and *g*. In random networks with size 100 and different average degree 〈*k*〉. The Absolute value of correlation coefficient is high in all cases except in cases when the value of the input signal is very small. ${\varvec{U}} = - 100,{ } - 10,{ } - 1,{ } - 0.1,{ }0.1,{ }1,{ }10,{ }100$ are the input signal. The input signal is applied to all nodes (**a)** in 5 Consecutive steps, (**b)** in 20 Consecutive steps, (**c)** Single signal applied to the whole nodes with *m* = 5, and (**d)** Single signal applied to the whole nodes and *m* = 20*.* The graphs show the average value with bars corresponding to the standard deviation over 20 realizations.

**Figure 4:**
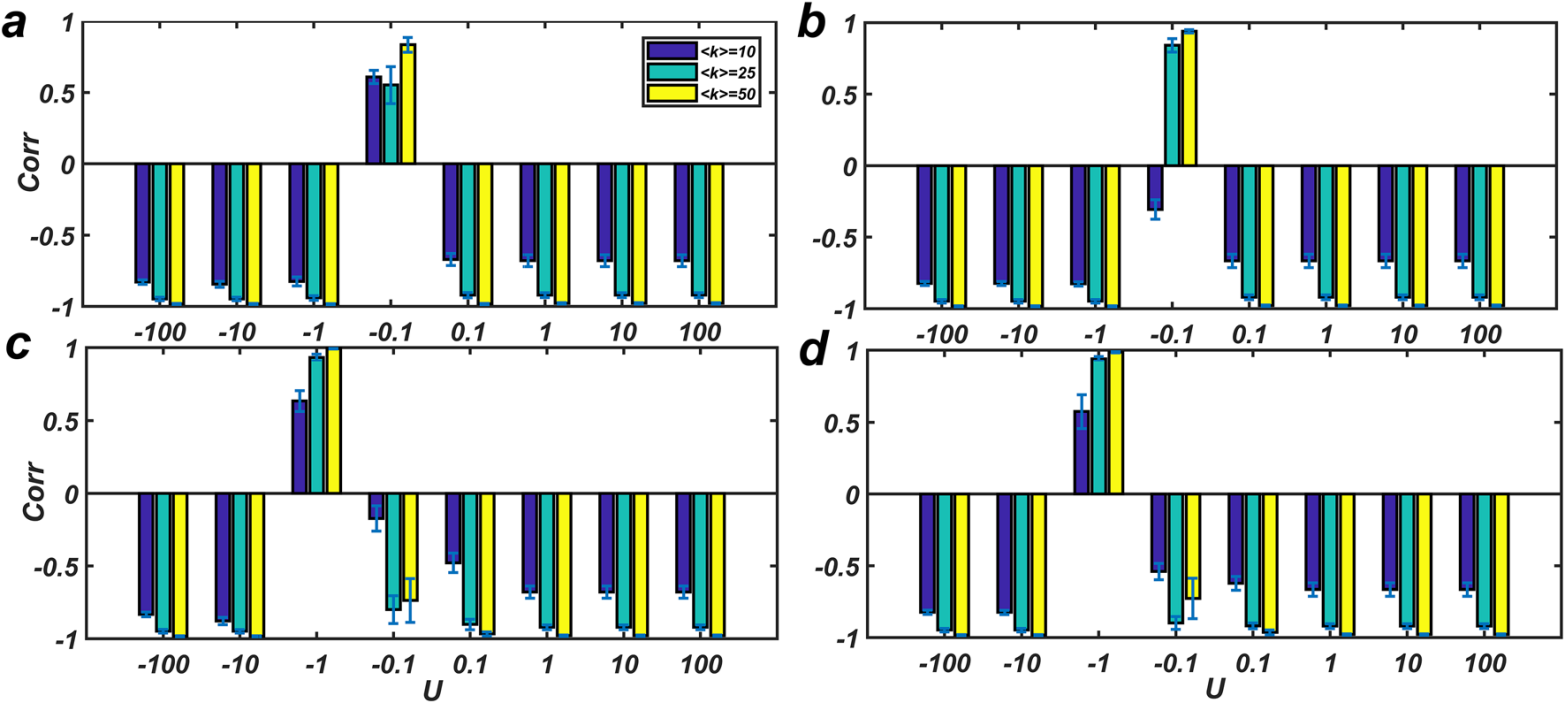
The correlation coefficient between two functions *f* and *g* when the input signal is applied to only drivers. Although the correlation is still negative, its absolute value is less compared to the case of applying the input signal to all nodes. Designations are as Fig. 3.

A directed network *G*(*A*) (with 100 nodes), in which *A* has 100 distinct eigenvalues and ${\text{det}}\left( A \right) \ne 0,$ was randomly generated with different average degrees of 10, 25 and 50. To prevent an increased state of the network, the weight of edges was considered $\pm 0.01,$ and the state of each node was initially set one. Then, an interval with 100 steps was considered, and signals with size 1 were applied to the network at times 0, 20 ,40, 60, 80 and 100. Based on the algorithm that we have provided in order to find the driver nodes with the minimal mediator nodes^15^, the network driver nodes are specified and then applied these signals to either (i) all nodes, or (ii) only driver nodes. At any time from 1 to 100*,* signals with different sizes (${\varvec{U}} = - 100, - 10, - 1, - 0.1, 0.1, 1, 10,100$) were applied to the network. Then, the correlation coefficient between *f* and *g* was calculated based on both the single and continuous signals (*m* = 5 and 20). Figure 2 shows *f* and *g* in a sample network with an average degree of 10, and when an input signal with a value of − 100 was applied to all nodes in 20 consecutive steps Figure 3 (Fig. 4) show the correlation coefficient between *f* and *g* when the input signals are applied to all (driver) nodes. It is seen that when the control is applied to all nodes, the correlation takes a larger absolute value than the case when the control is applied to only driver nodes. Furthermore, as the network become denser (i.e. their average degree increases), the correlation becomes stronger.

### Results on real networks

**Figure 5:**
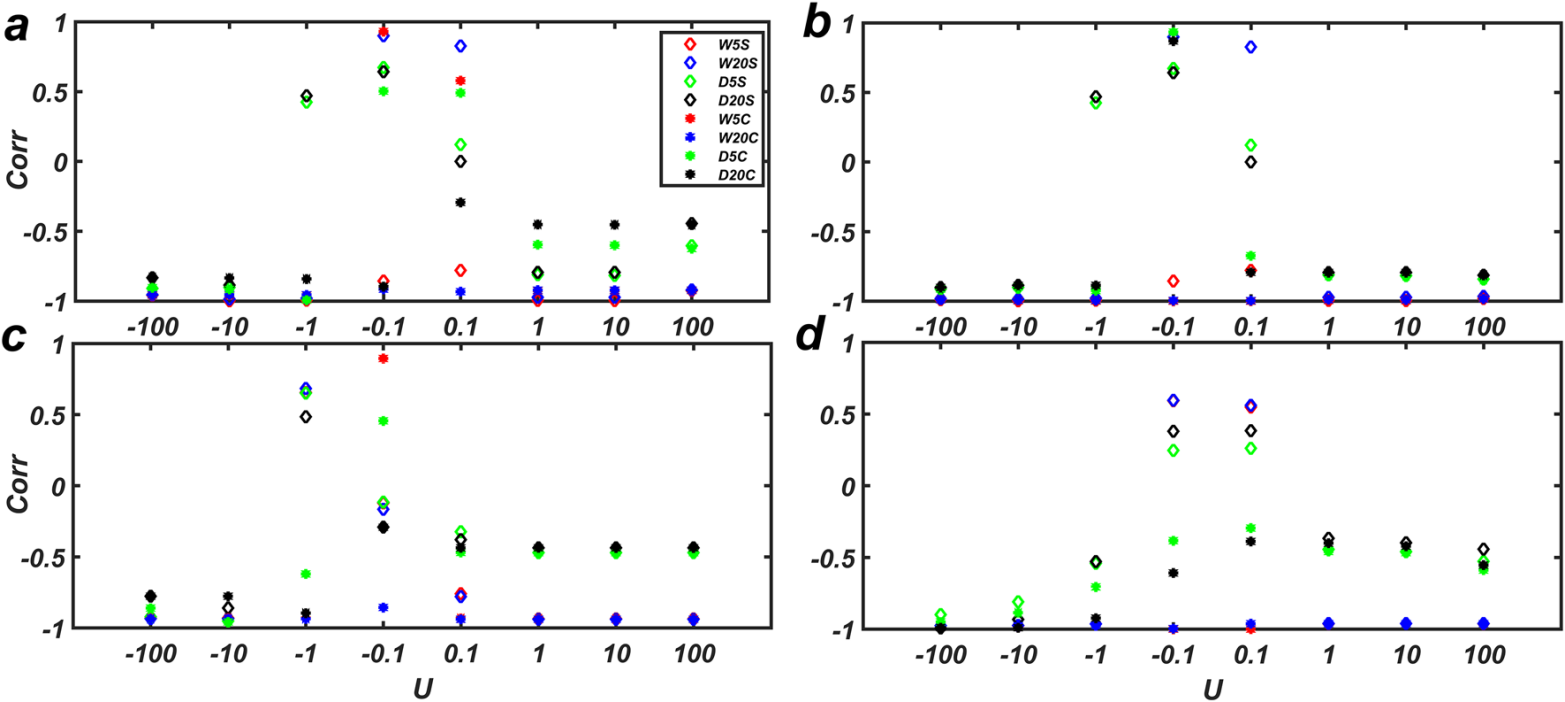
The correlation coefficient between *f* and *g* with different input signals in real networks. Designations and settings are as Table 1.

Like the previous section, the correlation coefficient between *f* and *g* at the given time intervals was calculated under different settings. The results obtained from ${\varvec{U}} = \pm 1$ were listed in Table 1 and those obtained from different sizes of ***U*** were presented in Fig. 5. The results obtained from different sizes of ***U*** show that the correlation coefficient between *f* and *g* is close to -1, except for the case of small-size input signal (${\varvec{U}} = \pm 0.1)$. Similar to synthetic networks, the correlation is stringer when the input signal is applied to all nodes rather than only drivers, which is somehow expected.

**Table 1:** The table shows the Pearson correlation coefficient between *f* (the strength of internal network fluxes) and *g* (the degree of the effectiveness of external signals) in different measurement modes, when input signals of different sizes are applied.

Name	Type	n	L	***U***	W5C	W20C	W5S	W20S	D5C	D20C	D5S	D20S
Mangrove^41^	Food Web	97	1492	1	− 0.9958	− 0.988	− 0.9933	− 0.9716	− 0.8144	− 0.7935	− 0.8101	− 0.7933
				− 1	− 0.9973	− 0.9933	− 0.9919	− 0.9787	− 0.9223	− 0.8848	0.4258	0.4709
*C. elegans*^42^	Neuronal	306	2345	1	− 0.9227	− 0.9199	− 0.9933	− 0.9716	− 0.5941	− 0.4498	− 0.8101	− 0.7933
				− 1	− 0.9565	− 0.9537	− 0.9919	− 0.9787	− 0.9916	− 0.8397	0.4258	0.4709
Prison inmate^43^	Trust	67	182	1	− 0.9356	− 0.9385	− 0.9355	− 0.9385	− 0.4693	− 0.4359	− 0.4693	− 0.4359
				− 1	− 0.9305	− 0.9339	0.6567	0.6851	− 0.6198	− 0.895	0.6524	0.4859
s420a^44^	Electronic circuits	252	399	1	− 0.9609	− 0.9617	− 0.9616	− 0.9603	− 0.4582	− 0.4004	− 0.4429	− 0.3661
				− 1	− 0.9748	− 0.9751	− 0.9634	− 0.9622	− 0.7044	− 0.9225	− 0.5399	− 0.5283

Definition of parameters: **n** is the number of nodes, **L** is the number of edges, ***U*** is the input signal applied to the network, which is considered to be 1 or − 1. The correlation coefficient between *f* and *g* is calculated in different settings as follows. including The input signal is applied to (W5C) all nodes in 5 Consecutive steps, (W20C) to all nodes in 20 Consecutive steps, (W5S) all nodes and the signal is applied at the first step and there are not any signals in the next 4 steps, (W20S) all nodes and the signal is applied at the first step and there are not any signals in the next 19 steps (D5C) the driver nodes in 5 Consecutive steps, (D20C) the driver nodes in 20 Consecutive steps, (D5S) the driver nodes and the signal is applied at the first step and there are not any signals in the next 4 steps, and (D20S) the driver nodes and the signal is applied at the first step and there are not any signals in the next 19 steps.

## Conclusions

A dynamical network is controllable if the state of its nodes can be steered from any initial value to a desired final value in a finite time. Often, external control signals are applied to a number of select nodes, called drivers, to control the networks. In some applications, it is critical to apply in control signals in appropriate time(s) to obtain the maximum control performance. If control signals are applied in appropriate times, the network might be controlled with smaller control signals, and thus less energy. Therefore, one may optimize the energy required for the control. In this manuscript, we proposed a methodology to final the best times to apply the external control signals to network nodes. We showed that applying the external signals to the network is most efficient when the value of positive and negative internal fluxes of the system is small. Under this condition, there would be no opposite forces in the system, and thus even small control signals could be effective.

## Methods

### State-variable response of linear systems

In a network with dynamics of motion as $\dot{\user2{X}}\left( t \right) = A{\varvec{X}}\left( t \right) + B{\varvec{U}}\left( t \right)$, if there are not any external signals, the state of the nodes will be obtained from the equation:

8 $${\varvec{X}}\left( t \right) = e^{At} {\varvec{X}}\left( 0 \right)$$

If an external signal is applied to the network, the state will be obtained using the following equation:

9 $${\varvec{X}}\left( t \right) = e^{At} {\varvec{X}}\left( 0 \right) + \mathop \smallint \limits_{0}^{t} e^{{A\left( {t - T} \right)}} B{\varvec{U}}\left( T \right)dT$$

### State transition matrix

The state of network nodes is obtained from the following equations:

10 $${\varvec{X}}\left( t \right) = e^{A} {\varvec{X}}\left( {t - 1} \right) + \mathop \smallint \limits_{t - 1}^{t} e^{{A\left( {t - T} \right)}} B{\varvec{U}}\left( T \right)dT = e^{A} \left( {{\varvec{X}}\left( {t - 1} \right) + \left( {\mathop \smallint \limits_{t - 1}^{t} e^{ - At} dT} \right) \times {\varvec{U}}} \right) = e^{A} \left( {{\varvec{X}}\left( {t - 1} \right) + A^{ - 1} \left( {I - e^{ - A} } \right) \times {\varvec{U}}} \right)$$

and $$e^{A} = M\hat{e}M^{ - 1}$$ which

11 $$M = \left[ {m_{1} \left| {m_{2} } \right| \ldots {|}m_{n} } \right] \;{\text{and}}\;\hat{e} = \left[ {\begin{array}{*{20}c} {\begin{array}{*{20}c} {e^{{\lambda_{1} }} } & 0 \\ 0 & {e^{{\lambda_{2} }} } \\ \end{array} } & {\begin{array}{*{20}c} 0 & 0 \\ 0 & 0 \\ \end{array} } \\ {\begin{array}{*{20}c} 0 & 0 \\ 0 & 0 \\ \end{array} } & {\begin{array}{*{20}c} \ldots & 0 \\ 0 & {e^{{\lambda_{n} }} } \\ \end{array} } \\ \end{array} } \right]_{n \times n}$$

$\lambda_{1} ,\lambda_{2} , \ldots ,\lambda_{n}$ and $m_{1} ,m_{2} , \ldots ,m_{n}$ are eigenvalues and eigenvectors of the matrix A, respectively.

#### Theorem

*In a Linear Time Invariant (LTI) system, the maximum effect of the input signals will be produced when the internal fluxes of the system are weak*.

#### *Proof*

If the dynamics of the systems are regarded as $\user2{ \dot{X}}\left( t \right) = A{\varvec{X}}\left( t \right) + B{\varvec{U}}\left( t \right)$, then

12 $${\varvec{X}}\left( t \right) = e^{At} {\varvec{X}}\left( 0 \right) + \mathop \smallint \limits_{0}^{t} e^{{A\left( {t - T} \right)}} B{\varvec{U}}\left( T \right)dT$$

The first part of the formula is derived from the initial conditions of the system, while the second part is derived from the input signals. So, the fraction

13 $$\frac{{\mathop \smallint \nolimits_{0}^{t} e^{{A\left( {t - T} \right)}} B{\varvec{U}}\left( T \right)dT}}{{e^{At} {\varvec{X}}\left( 0 \right)}}$$

specifies the effect of the input signals based on the initial conditions of the system. By removing $e^{At}$ from the fraction and the placement of the equation

14 $${\varvec{X}}\left( 0 \right) = A^{ - 1} \left( {\dot{\user2{X}}\left( 0 \right) - B{\varvec{U}}\left( 0 \right)} \right)$$

in the fraction, we will acquire the following equation:

15 $$\frac{{\mathop \smallint \nolimits_{0}^{t} e^{{A\left( {t - T} \right)}} B{\varvec{U}}\left( T \right)dT}}{{e^{At} {\varvec{X}}\left( 0 \right)}} = \frac{{\mathop \smallint \nolimits_{0}^{t} e^{ - AT} B{\varvec{U}}\left( T \right)dT}}{{{\varvec{X}}\left( 0 \right)}} = \frac{{\mathop \smallint \nolimits_{0}^{t} e^{ - AT} B{\varvec{U}}\left( T \right)dT}}{{A^{ - 1} \left( {\dot{\user2{X}}\left( 0 \right) - B{\varvec{U}}\left( 0 \right)} \right)}}$$

When the numerator of the fraction is constant, the maximum value will be obtained when the denominator of the fraction converges to zero. Thus, the value of $A{\varvec{X}}\left( 0 \right)$ will converge toward zero: ($A{\varvec{X}}\left( 0 \right) = \dot{\user2{X}}\left( 0 \right) - B{\varvec{U}}\left( 0 \right) \approx 0$) and this would be achieved when the internal fluxes of the system are weak.
